# Investigation of host and pathogen responses to estrogen in cystic fibrosis

**DOI:** 10.1186/1753-6561-7-S1-P4

**Published:** 2013-01-30

**Authors:** Moyser Mulla, G Lavelle, PJ McKiernan, CM Greene

**Affiliations:** 1 Dept. Medicine, Royal College of Surgeons in Ireland, Education and Research Centre, Beaumont Hospital, Dublin, Ireland

## Introduction

A ‘gender gap’ exists in Cystic Fibrosis (CF). Females acquire earlier microbial infections; have worse lung function and poorer survival rates [1]. The sex-hormone estrogen (estradiol, E2) has recently been highlighted as a key molecule responsible for the CF gender dichotomy [2]. *Pseudomonas aeruginosa* which colonises the CF lung and dominates at end stage disease undergoes mucoid conversion in response to E2 [2,3]. The aim of this project was to study other roles of E2 in host and pathogen responses by investigating its effects on the growth rate of *Ps. aeruginosa* and the expression of catalase and superoxide dismutase (SOD) in CF bronchial epithelial cells.

## Methods

Growth rate of *Ps. aeruginosa* (PA01) in the presence or absence of E2 was measured by recording optical density (OD _600nm_) at different time points and by calculating cfu/ml. Measurements of catalase and SOD gene expression in E2-treated CFBE410- airway epithelial cells were carried out using real time qRT-PCR. Results were analysed using Graphpad PRISM 5.0.

## Results

E2 had no effect on the growth of *Ps. aeruginosa* when compared to control. The expression of catalase mRNA in CFBE410- cells in response to E2 was not altered however, there was two-fold increase in SOD gene expression in response to 10 nM E2, 24hr (p= 0.0057).

## Conclusion

Estradiol has no effect on the growth of *Ps. aeruginosa in vitro.* In CF bronchial epithelial cells although catalase gene expression remains unchanged, E2 increases SOD expression, potentially increasing hydrogen peroxide levels and contributing to *Ps. aeruginosa* mucoid conversion.
